# Hemophagocytic Lymphohistiocytosis Triggered by Herpes Simplex Virus 1 and 2: A Narrative Review

**DOI:** 10.3390/hematolrep16030047

**Published:** 2024-07-26

**Authors:** Andria Papazachariou, Petros Ioannou

**Affiliations:** 1 Department of Internal Medicine, University Hospital of Heraklion, 71500 Heraklion, Greece; 2 School of Medicine, University of Crete, 71003 Heraklion, Greece

**Keywords:** HSV 1 and 2, HLH, epidemiology, mortality

## Abstract

**Introduction**: Hemophagocytic lymphohistiocytosis (HLH) is a rare, life-threatening syndrome characterized by an uncontrolled hyperinflammatory reaction. HLH is classified into primary (familial) and secondary (acquired). Secondary HLH is commonly triggered by infections, with viral infections being a leading cause. Its epidemiology and clinical features in cases associated with herpes simplex virus 1 and 2 remain underexplored. This study aimed to review all previously described cases of HSV-1 or -2-triggered HLH and provide information about this syndrome’s epidemiology, microbiology, clinical characteristics, treatment, and outcomes. **Methods**: A narrative review was performed based on a search in PubMed, the Cochrane Library, and Scopus. Studies published until 27 April 2024 providing relevant data for HLH due to HSV 1 and 2 in humans were included. **Results**: We identified 29 eligible studies reporting HLH due to HSV 1 and 2, involving 34 patients. Half of them were adults, and half were neonates. Fever and splenomegaly were the most common clinical findings. Most patients were diagnosed with HSV-1 (64.7%), with PCR being the primary diagnostic method. The median duration of in-hospital treatment was 21 days, with acyclovir and steroids being the mainstays of therapy. The overall mortality rate was 41.2%, and AST levels emerged as an independent predictor of mortality. **Conclusions**: Our findings underscore the need for heightened awareness surrounding HLH triggered by HSV 1 and 2 and the importance of prompt diagnosis and tailored treatment approaches.

## 1. Introduction

Hemophagocytic lymphohistiocytosis (HLH) is a rare, life-threatening clinical syndrome, marked by an unregulated hyperinflammatory reaction leading to uncontrolled activation of T-cells and macrophages, systemic inflammation, and subsequent tissue damage [1]. Its global epidemiology is unknown; however, in Japan, its prevalence is estimated to be 1:800,000 people; in China, 1.04:1,000,000 [2]; and in England, 1–2:1,000,000 [3]. The overall mortality rate fluctuates depending on the underlying cause, yet it commonly remains elevated, typically hovering around 40% [4].

HLH can be categorized into two main types: familial and acquired HLH. Acquired HLH, or secondary HLH, is triggered by infections, autoimmune diseases, malignancies, or immunosuppression [5,6,7,8]. Infectiously triggered HLH commonly arises from bacterial, viral, fungal, and parasitic infections, with viral infections being the predominant cause. Among viral infections, the Epstein–Barr virus (EBV) is most frequently implicated in adults [9], while herpes simplex virus (HSV) is commonly associated with neonates [10]. The diagnosis of HLH can be established if either A or B is fulfilled according to Table 1 [1,11].

The clinical presentation of HLH varies, but common findings typically include fever, hepatosplenomegaly, and cytopenias. According to the diagnostic criteria, the diagnosis of HLH requires the fulfillment of at least five out of eight specific criteria, which include fever; splenomegaly; cytopenias affecting at least two of three lineages in the peripheral blood; hypertriglyceridemia and/or hypofibrinogenemia; hemophagocytosis in the bone marrow, spleen, or lymph nodes; low or absent natural killer (NK) cell activity; hyperferritinemia; and high levels of soluble interleukin-2 receptor (sIL-2r) [12]. 

In the management of familial HLH, chemotherapy is typically utilized as a bridge to hematopoietic stem cell transplantation. Conversely, for acquired HLH, immunosuppressive therapy is the mainstay of treatment. This approach often involves the use of glucocorticoids, IL-receptor antagonists, cyclosporine, and etoposide, along with addressing the underlying trigger, such as acyclovir in cases associated with HSV infection [13]. When considering data on HLH triggered by HSV infection, a significant gap in the literature becomes apparent. Therefore, to provide a comprehensive overview of the existing evidence, this narrative review and meta-analysis was conducted to address the data concerning the epidemiology and mortality rates of HLH associated with HSV 1 or 2, along with insights into the diagnostic criteria and treatment strategies. 

## 2. Materials and Methods

**Figure 1:**
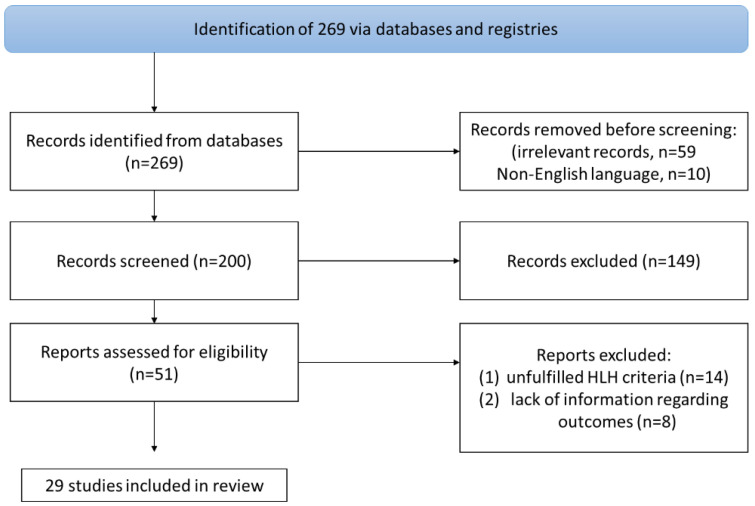
Flowchart of this study. HLH: Hemophagocytic lymphohistiocytosis.

This narrative review extracted and collected data regarding HLH syndrome associated with herpes simplex virus 1 and 2 in humans. The primary objective of the present study was to provide information regarding the epidemiology and the mortality of this infection. Presenting data on (a) the type of HSV, (b) the patients’ clinical characteristics, (c) the diagnostic criteria of HLH, and (d) their treatment was among the secondary outcomes of this study. For this review, the Scopus, PubMed/Medline, and Cochrane Library databases were searched for eligible articles reporting “Hemophagocyt* AND [HSV OR (herpes AND simplex AND virus)]” until 27 April 2024. Inclusion criteria for this narrative review included primary research papers such as observational studies, case reports, case series, and RCTs that provided data at least about epidemiology, clinical and microbiological criteria, and outcomes on HLH associated with HSV 1 or 2 infection in humans. Papers that were not in the English language were excluded. The exclusion criteria included experimental studies in animals, secondary research papers such as reviews and meta-analyses, and studies not referring to cases of HLH associated with HSV 1 or 2 in humans. Additionally, perspectives, editorials, and papers not reporting primary research results were excluded for further analysis. The remaining articles were examined following the snowball procedure to assess potential studies. The flowchart methodology of this research is available in Figure 1.

The collected data encompassed various aspects such as the publication year; country of origin; study design; demographic details of patients such as age and gender; relevant medical history (drug-induced or primary immunosuppression, autoimmune disease, existence of malignancy, previous history of HLH); clinical symptoms until diagnosis such as fever, jaundice, lymphadenopathy, mucocutaneous vesicles, and splenomegaly or/and hepatomegaly; laboratory values and microbiology values (type of HSV, method of identification, concomitant infection); treatments administered; and outcomes (i.e., complications, cure, or death). The association of mortality with HLH due to HSV 1 or 2 infection and the causal microbiology were reported according to the study authors. In each case, the diagnosis of HLH was confirmed by the current study’s investigators, based on the data provided by the authors in each study and the diagnostic guidelines for HLH presented by the Histiocyte Society in 2004, wherein five of the eight criteria should be fulfilled [12]. 

In the present review, the data are presented in numbers (%) for categorical variables and median values (IQR) for continuous variables. For the initial analysis regarding patient characteristics, symptoms, laboratory values, and the duration of hospitalization, the study population was stratified into two categories based on age: neonates and adults. Neonates and adults were compared regarding their outcomes (clinical cure or death). A comparative analysis was conducted between neonates and adults concerning their laboratory results. Continuous variables were compared using the Student *t*-test for normally distributed variables and the Mann–Whitney U-test for non-normally distributed variables. Categorical variables were compared using Fisher’s exact test due to the relatively small sample. A univariate logistic regression analysis explored factors associated with mortality, including sex, age, immunosuppression status, HSV type, laboratory results, number of diagnostic criteria, treatment, and duration of hospitalization. Subsequently, a multivariate logistic regression analysis was performed on variables that showed statistical significance. All of the above-mentioned statistics were calculated with SPSS version 25.0 (IBM Corp., Armonk, NY, USA). All tests were two-tailed, and *p*-values < 0.05 were considered statistically significant.

## 3. Results

### 3.1. Included Studies’ Characteristics

**Figure 2:**
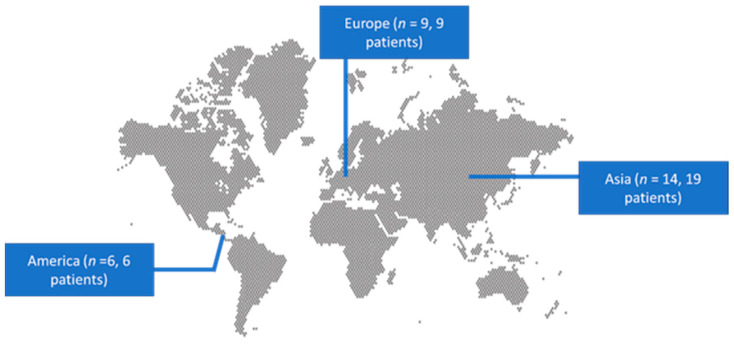
Geographical distribution of studies reporting hemophagocytic lymphohistiocytosis associated with HSV 1 and 2 worldwide.

A total of 269 articles, 188 from Scopus and 81 from PubMed, were screened. Finally, 29 met the present study’s inclusion criteria [13,14,15,16,17,18,19,20,21,22,23,24,25,26,27,28,29,30,31,32,33,34,35,36,37,38,39,40,41]. These 29 studies involved 34 patients in total. Among those studies, 14 were conducted in Asia, 9 in Europe, and 6 in North and South America. Figure 2 depicts the geographical distribution of HLH cases caused by HSV 1 and 2 worldwide. 

### 3.2. Epidemiology and Clinical Characteristics of HLH Associated with HSV 1 and 2

The study population with HLH associated with HSV 1 and 2 was 50% neonates (*n* = 17), and the rest (*n* = 17) were adults. The mean age for neonates was 9 ± 5 days, and the mean age for adults was 38 ± 18 years. Almost all neonates were diagnosed with HLH within the first week of their life. Regarding the age distribution among adults, approximately 64.7% were classified as young adults (18–30 years old), while the remaining 35.5% fell into the category of older adults (≥50 years old). Males comprised half of the study population. Data regarding immunosuppression were retrieved from 29 patients. Four patients (13.8%) had primary immunosuppression and four (13.8%) had drug-induced immunosuppression (one patient on steroids and the other three on a combination of steroids and azathioprine). Five patients (17.2%) had autoimmune disease, while none had malignancy. These five patients were exclusively adults rather than neonates (*p* = 0.05). Fever was evident in every adult participant (*n* = 17), unlike its presence in only 9 out of the 17 neonates (*p* = 0.03). Similarly, splenomegaly was detected in 12 adult patients compared to 6 neonates (*p* = 0.08). In terms of laboratory data, the median fibrinogen level was lower in neonates, at 75.5 mg/dL (52.5, 100), compared to that in adults at 160 mg/dL (104.8, 467.5) (*p* = 0.02). Ferritin levels were higher in neonates compared to adults, with medians of 58,770 ng/mL (40,000, 134,776) and 30,061 ng/mL (8529, 51,383), respectively (*p* = 0.05). Additionally, the prothrombin time (PT) in neonates was higher compared to that among adults, with medians of 80 (21,122) and 15.1 (4.2–22.9) sec, respectively. Triglyceride levels were higher in the adults, at 205.5 mg/dL (65.9, 350.8), compared to in the neonates, at 47 mg/dL (41, 76) (*p* = 0.04). Within the adult group, the median level of aspartate aminotransferase (AST) was notably elevated in individuals who died compared to those who experienced clinical cure: 11,306.5 U/L vs. 1807 U/L, respectively. The patients’ characteristics, symptoms on admission and during hospitalization, and laboratory values regarding HLH outcomes can be seen in Table 2 and Table 3.

### 3.3. Microbiology and Diagnosis of HLH Associated with HSV 1 and 2

In the present review, the prevailing type of HSV was HSV-1, in 22 cases (64.7%), while HSV-2 was detected in 9 cases (26.5%). Of the 22 cases with HSV-1, 13 were adults and 9 were neonates. Data on HSV type were absent for three patients. The predominant method for HSV identification was a Polymerase Chain Reaction (PCR) analysis of peripheral blood, utilized in 25 patients (86.2%), followed by serological testing, which was utilized in 7 patients (24.1%). In these patients, a primary HSV infection was identified, as seroconversion for IgM and IgG anti-HSV antibodies was observed during hospitalization. Eight patients (23.5%) underwent a combined analysis of their peripheral blood and cerebrospinal fluid (CSF) via PCR analysis, while immunohistochemistry in conjunction with PCR was conducted in four patients (11.8%). The median HSV DNA copy number detected via PCR in peripheral blood was 160 × 10^5^ (3.32, 160), and this was 4250 (1075, 5625) in the CSF samples.

**Figure 3:**
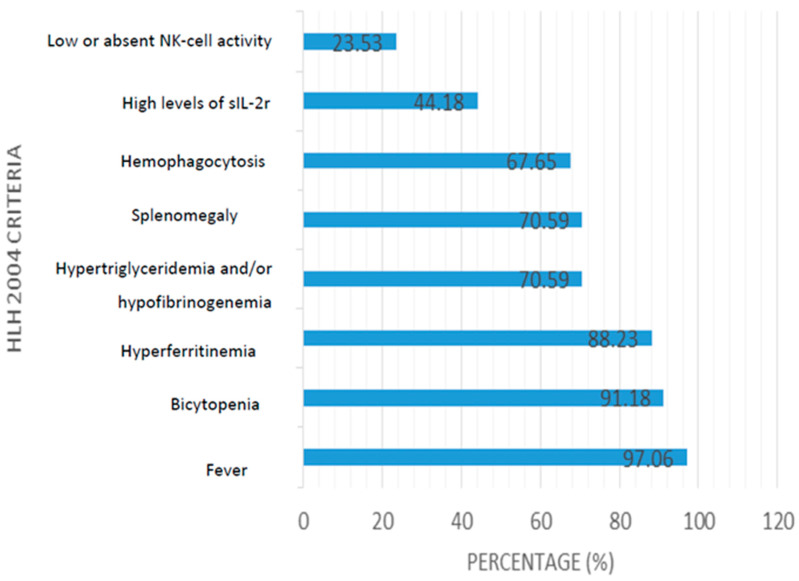
Frequency of criteria fulfilled for the diagnosis of HLH associated with HSV 1 and 2.

Regarding the fulfillment of diagnostic criteria for HLH, 21 individuals (61.8%) met five criteria, with 6 individuals (26.5%) meeting six criteria. Solely one patient fulfilled all eight criteria. Genetic testing to rule out familial HLH in neonates was conducted only in two studies. The mutation analysis of the genes responsible for familial HLH included PRF1, UNC13D, STX11, and STXBP2 [14,15]. Fever was the most prevalent criterion, noted in 33 individuals (97.1%), followed by bi-cytopenia in 31 patients (91.2%), hyperferritinemia in 30 (88.2%), splenomegaly in 24 (70.6%), and hypertriglyceridemia and/or hypofibrinogenemia in 24 (70.6%). Additional criteria included hemophagocytosis in bone marrow (67.6%), elevated sIL-2R levels (44.1%), and low or absent NK cell activity (23.5%). Upon conducting a Student t-test to compare the number of HLH criteria between surviving and deceased individuals, no statistically significant differences were discerned. Figure 3 depicts the frequency of criteria fulfilled for the diagnosis of HLH in all cases. 

### 3.4. Treatment and Outcomes of HLH Associated with HSV 1 and 2

The treatments administered to patients with HLH caused by HSV 1 and 2 are described in detail in Table 4. Among the survivors, the median duration of in-hospital treatment was 21 days (20, 33), and the total treatment duration was 24 days (14, 63). Initially, empiric antibiotic therapy was administered to 18 individuals (52.9%). All patients received acyclovir. Additional treatment included steroids in 28 patients (82.4%), intravenous immunoglobulin (IVIG) in 15 patients (44.1%), etoposide in 12 patients (35.3%), cyclosporine-A in 9 patients (26.5%), foscarnet in 2 patients (5.9%), emapalumab in 1 patient (2.9%), anakinra in 1 patient (2.9%), and tocilizumab in 1 patient (2.9%). Plasma exchange was needed for five patients (14.7%). 

Concomitant infections were documented in three patients, including one with bacteremia, one with aspiration pneumonia, and one with gastrointestinal infection. Complications were reported in 24 individuals (70.6%), with no data available for 4 cases. Organ dysfunction was observed in 19 patients (55.9%); 7 patients experienced multi-organ failure, while 5 individuals (14.7%) presented with a combination of hepatic and respiratory failure. Shock was present in eight patients (23.5%): seven of them died (87.5%), whereas one (12.5%) experienced clinical cure (*p* = 0.010). Disseminated intravascular coagulation (DIC) was reported in seven patients (20.6%), neurological manifestations in five patients (14.7%), and sepsis in three patients (8.8%). Fifteen patients (44.1%) required admission to an intensive care unit. The overall mortality rate was 41.2% (14 patients), and the median duration of hospitalization for those who died was 7 days (2.5, 21.5).

### 3.5. Statistical Analysis of HLH Associated with HSV 1 and 2

In the univariate regression analysis, a range of parameters, including sex, age, the presence of immunosuppression, HSV type, laboratory results, diagnostic criteria, treatments, and duration of hospitalization, was investigated. This analysis revealed a negative correlation between platelet count (Pearson’s r = −0.466, *p* = 0.012) and mortality. Conversely, lactate dehydrogenase (LDH) levels (Pearson’s r = 0.558, *p* = 0.025) and AST levels (Pearson’s r = 0.643, *p* = 0.001) showed positive correlations with mortality. However, in the multivariate logistic regression analysis, only AST emerged as an independently associated factor influencing mortality (*p* = 0.039).

## 4. Discussion

This narrative review comprehensively discusses the epidemiological and clinical features of patients diagnosed with HLH due to HSV 1 and 2, encompassing diagnostic criteria alongside treatment options. Both groups (neonates and adults) are represented equally. Eight out of thirty-four individuals were immunocompromised due to either primary disease or drug-induced immunosuppression. The most common clinical presentations included fever, hepatomegaly, splenomegaly, and mucocutaneous vesicles. Acyclovir and steroids, alone or in combination, were the most frequent treatment options. 

HSV type 1 and HSV type 2 are members of the Herpesviridae family and are the causative agents of oral and genital herpes, respectively. HSV infections are among the infections most frequently encountered by humans [42]. Both viruses share a similar pathogenesis, initiating infection through mucosal surfaces or abraded skin, leading to local viral replication and subsequent spread to sensory neurons. HSV infections can present in various clinical forms, ranging from asymptomatic to severe manifestations such as gingivostomatitis, keratoconjunctivitis, encephalitis, and neonatal herpes [43]. The fact that certain individuals are more prone than others to develop a severe disease upon HSV infection can be partially explained by the existence of genetic polymorphisms in humans [44]. The clinical course of HSV infections includes both lytic and latent phases. During the lytic phase, active viral replication occurs, resulting in cell lysis and the characteristic lesions. Following the initial infection, HSV establishes latency in sensory ganglia, where the viral genome persists in a dormant state. Periodic reactivation can occur, leading to recurrent infections that can be triggered by factors such as stress, immunosuppression, or other infections [45,46].

The age distribution of the adult population in this review aligns with the population pyramid distribution observed in a previous meta-analysis of 16,136 adult patients diagnosed with HLH, as there are peaks in both young adults and older adults [47]. Although the literature suggests malignancy and autoimmune diseases as the most frequent underlying triggers and associated conditions, in these reviews, no one presented with malignancy, whereas one in five patients presented with an autoimmune disease. This can be explained by the fact that this review studied a small subset of HLH-associated-with-HSV cases. One in three individuals presented with immunosuppression [48,49]. Furthermore, this review included cases from Asia, Europe, and America, while there is a lack of representation for other large-population continents, like Africa. However, recent works in the literature indicate an increase in observational studies on HLH within African populations [50,51].

Regarding clinical manifestations, fever, mucocutaneous vesicles, and splenomegaly were predominant in adult patients with HLH; conversely, respiratory symptoms were more apparent in neonates. The high incidence of fever and splenomegaly observed in our study closely aligns with findings from other studies. For instance, in a review encompassing 18 studies focusing on adults with HLH caused by infectious diseases, particularly EBV and Cytomegalovirus (CMV), fever was highlighted as a primary symptom, likely attributable to the excessive production of IL-1 [52]. Similarly, in another review focusing on cases of HLH, splenomegaly was reported at higher rates in adults compared to children, mirroring the findings of our study [46]. According to the existing literature regarding neonates, the occurrence of multi-organ dysfunction, such as respiratory distress and renal or liver dysfunction, is prevalent. Notably, in one review analyzing cases of neonates with HLH, a high incidence of required mechanical ventilation was reported [10].

As for laboratory data, fibrinogen levels were observed to be lower in neonates, while neonate ferritin levels were higher compared to those in adults. This evidence aligns with the findings from a review that compared variables between children and adults with HLH; however, no statistical significance was found in that review [53]. Prothrombin time (PT) values were found to be higher in neonates in their first week of life, which may be related to vitamin K deficiency rather than HLH [54]. Moreover, elevated AST levels in the adult population were found to be more pronounced in individuals who died. The literature varies regarding the levels of liver enzymes in HLH, despite liver injury being a common complication. Most studies indicate mild to moderate elevation in AST levels in HLH, with liver failure being rare [55,56]. On the other hand, other studies in patients with HLH have demonstrated higher AST levels, which were associated with increased mortality [57,58,59]. Additionally, a recent retrospective study in pediatric HLH patients revealed a correlation between higher AST levels and mortality [60]. Nevertheless, these studies encompassed patients with various causes of secondary HLH, not solely viral-associated HLH, thus necessitating further research to yield more definitive conclusions. 

Most patients were diagnosed with HSV-1, with higher rates observed in adults. This differs from the literature, which commonly identifies HSV as the most prevalent infection in neonates and EBV as the most prevalent in adults [10,49]. As PCR is the most common molecular diagnostic technique [61], a PCR analysis of peripheral blood was frequently used to confirm the presence of HSV in the majority of cases in this review. Notably, in almost all cases of virus-triggered HLH, the type of virus was diagnosed using PCR analysis [62,63]. Consequently, routine PCR testing for common viruses associated with HLH should be considered for all HLH patients [64].

Diagnosing HLH remains challenging and requires a high index of suspicion. This is due to the diverse presentations among patients, with many often meeting more than five criteria later in the progression of the disease rather than in its early stages [65,66]. Another hurdle is the differential diagnosis with disseminated HSV, as patients often exhibit similar clinical symptoms such as fever, splenomegaly, and hyperferritinemia [67,68,69]. As illustrated in the flowchart, 14 cases were excluded because they did not fulfill diagnostic criteria or presented with disseminated HSV infection, making it difficult to distinguish from HLH, despite these patients being treated as HLH. In our cases, two criteria out of the eight were almost always positive: fever and bi-cytopenia. When compared to other HLH-secondary-to-viruses cases such as HIV [70], CMV [71,72], Ebola [73], and EBV [62], fever emerges as the most prevalent criterion for diagnosing HLH. Cytopenias serve as crucial laboratory markers for virus-triggered HLH [70,71,73,74]. Another criterion with a high positivity rate was hyperferritinemia (ferritin ≥ 500 μg/L). Although hyperferritinemia lacks specificity for HLH, it should be promptly considered for HLH in individuals with disseminated HSV infection and significantly elevated ferritin levels [15,37,67]. Furthermore, although hepatosplenomegaly is a common finding in HSV infection, splenomegaly in cases with HLH-HSV was observed in 70% of patients, compared to 100% in HLH–human immunodeficiency virus (HIV) [70] and HLH-CMV [71]. Reduced NK cell activity was found in one-fifth of our cases. Even though NK cells play a pivotal role in modulating the immune response and their activity is decreased in HLH [75], they are typically analyzed in specialized laboratories; therefore, the fulfillment of this criterion typically depends on factors such as the availability of specialized equipment and expertise [76]. A diagnosis of HLH can be assumed because of findings such as hemophagocytosis in bone marrow; however, this is neither sensitive nor specific [77]. The present study found hemophagocytosis in bone marrow in more than half of cases. Higher rates were observed in other infection-associated HLH [70,71,78,79]. However, as previously mentioned, a negative result does not rule out the diagnosis: in some viruses, such as Ebola–HLH, no hemophagocytosis has been reported [73]. 

Both sporadic and familial cases of HLH can be initiated by infectious causes; yet, distinguishing them is crucial for HLH diagnosis, as non-infection-associated HLH represents an important part of HLH [48]. Familial HLH, unlike secondary HLH, is a disorder of early childhood [80]. However, coincident infection will not differentiate between inherited disease and acquired disease [81]. The role of HLH-related genes and the impact of their mutations on the frequency of HLH are important considerations. Genetic mutations associated with HLH—such as those in familial HLH (PRF1, UNC13D, and STX11 genes), several granule/pigment-abnormality genes (RAB27A, LYST, AP3B1), X-linked lymphoproliferative disease genes (SH2D1A, XIAP), and others such as nlrc4 and cdc42—can significantly influence an individual’s susceptibility to developing HLH in response to viral infections. Their mutations often result in defects in cytotoxic function and immune regulation, leading to an exaggerated immune response upon encountering triggers such as viruses [82,83,84]. Yet, only two studies in neonates proceeded to genetic testing in order to rule out familial causes of HLH. On the other hand, malignancy, particularly hematologic malignancy, is the most common trigger identified in adults with HLH, compared to this being a relatively rare event in the pediatric population [85]. The activation of NK cells, lymphocytes, and macrophages and the secretion of high levels of cytokines and chemokines are common between cases of HLH; however, variations in the initiating pathophysiological mechanisms do exist between infection- and non-infection-associated HLH. Familial HLH is caused by mutations in genes implicated in granule-mediated cytotoxicity, impairing the function of NK and CD8+ cytotoxic T-lymphocytes (CTLs), or can develop as a complication in X-linked lymphoproliferative disease [86]. It has been suggested that heterozygous variants in genes related to the pathway of immune response contribute to the development of secondary HLH [87]. In the case of infection-associated HLH, the initiating mechanisms that lead to a defect in cytotoxicity and a poorly controlled immune response have not been completely elucidated and they are likely multifactorial. It is believed that immunological dysregulation is induced by viruses such as the herpes viruses through the infection of T-cells, leading to an excessive production of TNF-a and IFNγ, increased macrophage activation, sustained activation of CTLs, and, finally, aggressive hyperinflammatory syndrome [88]. Regarding treatment, since the pathophysiology of infection-associated HLH involves the direct viral effect and the immune system’s overactivation, antiviral therapy alongside immunosuppressive treatment is necessary. In contrast, non-infection-associated HLH, such as familial HLH, requires hematopoietic stem cell transplantation, and treating other secondary HLH involves treatment of the underlying condition, such as chemotherapy for malignancies or immunosuppressive therapy for autoimmune diseases, in addition to standard HLH protocols [1].

The conventional therapy for individuals with infection-induced HLH, except from antibiotic/antiviral treatment, is an immunosuppressive regime involving corticosteroids, either combined with etoposide or not [1,89]. The primary objective is to reduce circulating cytokines, as HLH stems from the overproduction of inflammatory mediators [52]. A combination of conventional therapy with cyclosporine A has also shown positive outcomes [90]. In the present review, all patients received acyclovir as an antiviral treatment for HSV, which in most cases was combined with steroids. Approximately one-third of the population received etoposide, with one-fourth receiving cyclosporine A. In select cases, supplementary immunosuppressive therapies were introduced, including interleukin receptor antagonists (IL-1Ra, IL-6Ra) or interferon-gamma (IFNγ)-blocking antibodies. Further investigation is necessary to identify optimal initial therapies. For instance, rituximab has shown promise in EBV-HLH [91]. In cases of infection-triggered HLH, the consideration of high-dose IVIG alongside steroids is recommended, as observed in almost half of our study’s patients. Notably, a personalized treatment approach, incorporating factors such as immunosuppression status, clinical presentation, laboratory results, and the specific viral infection type, is essential for optimal patient management [92]. Additionally, supportive care measures, including ICU transfer for organ support (mechanical ventilation, plasma exchange, vasoactive drugs), should not be overlooked [93]. 

It is worth noting that virus-triggered HLH is a rare condition with a poor prognosis [94]. The overall mortality rate in our study was determined to be 40%. Case fatality rates for infection-associated HLH have been reported to range from 41% to 75% depending on underlying diseases, age, and so on [95,96]. 

## 5. Conclusions

This study provides information about HSV-triggered HLH and summarizes its epidemiology, diagnostic criteria, treatment, and outcomes. However, it has certain limitations that warrant acknowledgment. It exclusively incorporates articles published in English and the included research is confined to primary research papers, potentially overlooking pertinent data from studies published in other languages and secondary sources such as meta-analyses or reviews. Moreover, the relatively small number of included studies may limit the generalizability of the findings. 

## Figures and Tables

**Table 1:** HLH diagnostic criteria.

A	Molecular diagnosis consistent with HLH [pathologic mutations of Perforin (PRF1), SH2D1A/SAP, UNC13D, Syntaxin 11 (STX11), MUNC18-2, Ras-related protein Rab27a (RAB27a)]	
B	Any 5 of the 8 following clinical and laboratory criteria for HLH	
	Fever	>38.5 °C
	Splenomegaly	
	Cytopenia (affecting ≥ 2 of 3 lineages in peripheral blood)	Hemoglobin < 9 g/dL (in infants < 4 weeks: Hb < 100 g/L) Platelets < 100 × 10^9^/L Neutrophils < 1.0 × 10^9^/L
	Hypertriglyceridemia and/or hypofibrinogenemia	Fasting triglycerides > 3.0 mmol/L (>265 mg/dL) Fibrinogen ≤ 1.5 g/L
	Hemophagocytosis in bone marrow, spleen, liver, lymph nodes, or other tissues	
	Low or absent natural killer cell activity	
	Serum ferritin concentration	≥500 μg/L
	Soluble CD25 (soluble IL-2 receptor)	≥2400 U/mL

HLH: Hemophagocytic lymphohistiocytosis.

**Table 2:** Neonate patients’ characteristics, symptoms, and laboratory values regarding HLH outcomes.

	Survived (*n* = 9)	Died (*n* = 8)	*p*-Value
Patient characteristics (neonates, *n* = 17):			
Age, median (IQR)	5 (4, 9)	5.5 (3.5, 9.75)	1.00
Male sex, *n* (%)	4 (44.4)	3 (37.5)	1.00
Primary immunosuppression, *n* (%)	0	1 (20.0)	0.42
Symptoms:			
Fever, *n* (%)	6 (66.7)	3 (37.5)	0.35
Weight loss, *n* (%)	0	2 (66.7)	0.40
Jaundice, *n* (%)	2 (22.2)	1 (12.0)	1.00
Hepatomegaly, *n* (%)	6 (66.7)	2 (25.0)	0.15
Splenomegaly, *n* (%)	4 (44.4)	2 (25.0)	0.62
Lethargy/drowsiness, *n* (%)	1 (11.1)	2 (25.0)	0.58
Mucocutaneous vesicles, *n* (%)	2 (22.2)	0	0.47
Skin rash, *n* (%)	1 (11.1)	0	1.00
Respiratory symptoms (tachypnea, dyspnea, apnea), *n* (%)	1 (11.1)	3 (37.5)	0.29
Median laboratory values (IQR):			
Leukocytes (K/mL)	4000 (2650, 8875)	1200 (700, -)	1.00
Hemoglobin (g/dL)	10.7 (8.6, 13.9)	8.1 (7.0, -)	0.17
Platelets (×10^3^ K/mL)	96 (39, 104)	17 (14, 65)	0.24
AST (U/L)	3543 (2055, 3804)	6966.5 (2439.5, 7403)	0.24
ALT (Ul/L)	943 (308, 1226)	2214 (539, 3127.5)	1.00
LDH (U/dL)	6805 (4765.5, 85.3)	8073 (3750, -)	1.00
Triglycerides (mg/dL)	63 (40.5, 158.5)	42.5 (41, -)	0.43
CRP (mg/dL)	1.7 (0.84, 6.5)	3.6 (1.7, 15.4)	1.00
Fibrinogen (mg/dL)	61 (43, 104)	92 (60, 98)	1.00
Ferritin (ng/mL)	40,604.5 (14,387.8, 77,771.5)	113,200 (76,650, 216,210)	0.70
sIL-2R (U/mL)	3205 (2472.5, 4730.5)	2503.5 (1068.3, 3979.3)	1.00
NK cell activity (%)	10 (0, -)	4 (4, 4)	1.00
PT (s)	80 (13.1, -)	74.7 (29.4, -)	1.00
Duration of hospitalization, median (IQR)	42 (27.8, 70)	21 (6, 39)	0.29

ALT: alanine transaminase; AST: aspartate transaminase; CRP: c-reactive protein; IQR: interquartile range; LDH: lactate dehydrogenase; NK: natural killer; PT: prothrombin time; sIL-2R: soluble interleukin 2 receptor.

**Table 3:** Adult patients’ characteristics, symptoms, and laboratory values regarding HLH outcomes.

	Survived (*n* = 11)	Died (*n* = 6)	*p*-Value
Patient characteristics (adults, *n* =17):			
Age, median (IQR)	36 (19, 59)	31.5 (25.5, 51.5)	1.00
Male sex, *n* (%)	6 (54.5)	4 (66.7)	1.00
Primary immunosuppression, *n* (%)	3 (27.3)	0	0.52
Drug-induced immunosuppression, *n* (%)	2 (18.2)	2 (33.3)	0.58
Autoimmune disease, *n* (%)	3 (27.3)	2 (33.3)	1.00
Symptoms:			
Fever, *n* (%)	11 (100)	6 (100)	
Jaundice, *n* (%)	0	2 (33.3)	0.11
Hepatomegaly, *n* (%)	7 (63.6)	2 (33.3)	0.34
Splenomegaly, *n* (%)	7 (63.6)	5 (83.3)	0.60
Lethargy/drowsiness, *n* (%)	3 (27.3)	1 (16.7)	1.00
Mucocutaneous vesicles, *n* (%)	5 (45.5)	1 (16.7)	0.33
Skin rash, *n* (%)	2 (18.2)	1 (16.7)	1.00
Abdominal pain (GI symptoms), *n* (%)	4 (36.4)	0	0.24
Median laboratory values (IQR):			
Leukocytes (K/mL)	1420 (850, 1600)	1360 (822.5, 1775)	1.00
Hemoglobin (g/dL)	10.7 (8.6, 13.9)	7.5 (6.7, 10.4)	1.00
Platelets (×10^3^ K/mL)	81 (47.5, 1125)	25.5 (14.8, 71.8)	0.61
AST (U/L)	1807 (1398, 4000)	11,306.5 (8166, 12,554.5)	0.03
ALT (Ul/L)	1803.5 (751, 3331.5)	3076 (839, 4819.5)	0.57
LDH (U/dL)	2284 (1345, 4248)	11,100 (6000, -)	0.17
Triglycerides (mg/dL)	281 (128.8, 502.3)	79 (61.6, -)	1.00
CRP (mg/dL)	9.8 (5.7, 10.4)	2.5 (1.8, -)	1.00
Fibrinogen (mg/dL)	29,726 (5296.8, 62,427)	140 (99, -)	1.00
Ferritin (ng/mL)	29,726 (5296.8, 62,427)	30,524 (9264.5, 6200)	0.85
sIL-2R (U/mL)	4590 (1574.5, 12,428)	7214.5 (3075, 20,867)	1.00
NK cell activity (%)	2.4 (2.4, 2.4)	-	-
PT (s)	15.1 (10.8, -)	13.02 (2.04, -)	0.88
Duration of hospitalization, median (IQR)	21 (14, 57.5)	6 (4, 12.5)	0.30

ALT: alanine transaminase; AST: aspartate transaminase; CRP: c-reactive protein; IQR: interquartile range; LDH: lactate dehydrogenase; NK: natural killer; PT: prothrombin time; sIL-2R: soluble interleukin 2 receptor: GI: gastrointestinal symptoms.

**Table 4:** Characteristics of patients in the included studies.

First Author(s)	Age	Sex	Type of HSV	HLH Criteria	Complications	Need for ICU	Treatment(s) Administered	Outcome
Alidjinou et al., 2015 [16]	59 years	Male	Hsv1	5	No	N/A	ACV, etoposide	Clinical cure
Cheney-Peters and Weber, 2019 [17]	65 years	Male	Hsv1	6	No	N/A	ACV	Clinical cure
Cusini et al., 2010 [18]	57 years	Female	Hsv1	5	Yes	Yes	ACV, steroids, etoposide, IVIG, platelets and erythrocyte replacement	Clinical cure
Drori et al., 2015 [19]	50 years	Male	Hsv1	5	Yes	Yes	ACV, etoposide, FFP, doxycycline/ceftazidime, hemodialysis	Death
Freytag et al., 2022 [20]	19 years	Female	Hsv1	5	Yes	Yes	ACV, steroids, foscarnet, IVIG, tocilizumab	Clinical cure
Grabovac et al., 2012 [11]	36 years	Male	Hsv1	5	Yes	Yes	ACV, steroids, IVIG, etoposide, FFP, blood transfusion, albumin, G-CSF, broad-spectrum antibiotics, vasopressors	Death
Honsig et al., 2017 [21]	21 years	Male	Hsv1	5	Yes	Yes	ACV	Death
Halstead et al., 2016 [22]	5 days	Female	Hsv2	5	Yes	Yes	ACV, steroids, vasopressors	Death
Ikumi et al., 2016 [23]	56 years	Male	Hsv2	6	Yes	N/A	ACV, steroids	Death
Kojima et al., 2012 [24]	3 days	Male	Hsv1	5	Yes	No	ACV, steroids, CsA	Clinical cure
Kurosawa et al., 2019 [25]	46 years	Male	Hsv2	5	No	No	Meropenem, ACV, steroids,	Clinical cure
Mckeone et al., 2021 [15]	11 days	Female	Hsv1	5	Yes	N/A	ACV, IVIG, steroids, etoposide, emapalumab, anakinra, FFP, hemodialysis	Death
Nagamura and Ishitobi, 2017 [26]	34 years	Female	Hsv1	5	Yes	N/A	Azathioprine, ACV, steroids, CsA, valacyclovir	Clinical cure
Nasser et al., 2018 [27]	36 years	Female	Hsv2	5	Yes	N/A	ACV, steroids	Clinical cure
Noh et al., 2015 [28]	14 years	Female	Hsv1	5	No	No	ACV, steroids	Clinical cure
Otsubo et al., 2016 [29]	6 days	Female	Hsv1	7	Yes	Yes	ACV, steroids, cefazolin, FFP, r-TM	Clinical cure
Saettini et al., 2021 [30]	18 years	Male	Hsv1	5	Yes	No	ACV, steroids, IVIG, ceftriaxone, amikacine, RBC transfusion	Clinical cure
Schwartz et al., 2019 [31]	27 years	Female	Hsv2	6	Yes	Yes	ACV, vasopressors	Death
Sonoda et al., 2019 [14]	6 days	Male	Hsv2	7	Yes	Yes	ACV, steroids, CsA, etoposide, IVIG, plasma exchange	Death
	4 days	Male	Hsv2	5	Yes	Yes	ACV, steroids, cyclosporin, IVIG	Clinical cure
Spinner et al., 2016 [32]	18 days	Female	Hsv1	6	Yes	N/A	ACV, steroids, vasopressors, vancomycin, meropenem, levofloxacin, liposomal amphotericin B	Death
States and Kapp, 2022 [33]	27 years	Female	Hsv1	5	Yes	Yes	ACV, steroids, etoposide, vasopressors	Death
Suzuki et al., 2009 [34]	5 days	Female	Hsv1	7	N/A	N/A	ACV, IVIG, steroids, CsA, etoposide	Death
	3 days	Male	Hsv1	6	Yes	N/A	ACV, IVIG, steroids, CsA, etoposide	Death
	8 days	Male	N/A	5	N/A	N/A	ACV, IVIG, steroids, CsA, etoposide	Clinical cure
	6 days	Female	N/A	6	N/A	N/A	ACV, IVIG, steroids, etoposide	Death
	4 days	Female	N/A	5	N/A	N/A	ACV, IVIG, steroids, CsA, etoposide	Clinical cure
Takagi et al., 2023 [35]	17 days	Female	Hsv1	6	Yes	N/A	ACV, steroids	Clinical cure
Takehara et al., 2019 [36]	2 days	Male	Hsv1	5	Yes	Yes	ACV, steroids, foscarnet, CRRT, vasopressors	Death
Vladescu et al., 2015 [37]	10 days	Female	Hsv2	6	Yes	Yes	ACV, ampicillin, gentamicin	Clinical cure
Wada et al., 2011 [38]	5 days	Female	Hsv2	5	Yes	N/A	ACV, IVIG, antibiotics	Clinical cure
Yabushita et al., 2017 [39]	69 years	Male	Hsv1	5	No	N/A	ACV	Clinical cure
Yamada et al., 2008 [40]	4 days	Male	Hsv1	6	Yes	Yes	Platelet replacement, FFP, ACV, steroids, CsA, IVIG, vasopressors	Clinical cure
Zhang et al., 2023 [41]	19 years	Male	Hsv1	8	Yes	Yes	ACV, steroids, etoposide, IVIG, imipenem	Clinical cure

FFP: fresh frozen plasma; ACV: acyclovir; IVIG: intravenous immunoglobulin therapy; CRRT: continuous renal replacement therapy; CsA: cyclosporine A; r-TM: recombinant thrombomodulin alfa, N/A: information not available.

## Data Availability

The data presented in this study are available on request from the corresponding author.

## References

[1] Janka G.E., Lehmberg K. (2013). Hemophagocytic Lymphohistiocytosis: Pathogenesis and Treatment. Hematology.

[2] Yao S., Wang Y., Sun Y., Liu L., Zhang R., Fang J., Jin R., Yu J., Li F., Bai J. (2021). Epidemiological Investigation of Hemophagocytic Lymphohistiocytosis in China. Orphanet J. Rare Dis..

[3] West J., Card T.R., Bishton M.J., Lanyon P., Ban L., Bythell M., Elliss-Brookes L., Manson J.J., Nanduri V., Rankin J. (2022). Incidence and Survival of Haemophagocytic Lymphohistiocytosis: A Population-based Cohort Study from England. J. Intern. Med..

[4] Hayden A., Park S., Giustini D., Lee A.Y.Y., Chen L.Y.C. (2016). Hemophagocytic Syndromes (HPSs) Including Hemophagocytic Lymphohistiocytosis (HLH) in Adults: A Systematic Scoping Review. Blood Rev..

[5] Imashuku S., Morimoto A., Ishii E. (2021). Virus-triggered Secondary Hemophagocytic Lymphohistiocytosis. Acta Paediatr..

[6] Setiadi A., Zoref-Lorenz A., Lee C.Y., Jordan M.B., Chen L.Y.C. (2022). Malignancy-Associated Haemophagocytic Lymphohistiocytosis. Lancet Haematol..

[7] Rajapakse P., Andanamala H. (2022). Hemophagocytic Lymphohistiocytosis Secondary to Immune Checkpoint Inhibitor Therapy. World J. Oncol..

[8] Yildiz H., Van Den Neste E., Defour J.P., Danse E., Yombi J.C. (2022). Adult Haemophagocytic Lymphohistiocytosis: A Review. QJM Int. J. Med..

[9] Yao S., He L., Zhang R., Liu M., Hua Z., Zou H., Wang Z., Wang Y. (2023). Improved Hemophagocytic Lymphohistiocytosis Index Predicts Prognosis of Adult Epstein-Barr Virus-Associated HLH Patients. Ann. Med..

[10] Balakumar N., Sendi P., Totapally B.R. (2022). Epidemiology and Outcomes of Neonatal Hemophagocytic Lymphohistiocytosis. Front. Pediatr..

[11] Grabovac V., Kardum-Peric M., Sokol S. (2012). Hemophagocytic Syndrome Associated with Human Herpesvirus I: A Case Report. Neurol.Croat..

[12] Henter J., Horne A., Aricó M., Egeler R.M., Filipovich A.H., Imashuku S., Ladisch S., McClain K., Webb D., Winiarski J. (2007). HLH-2004: Diagnostic and Therapeutic Guidelines for Hemophagocytic Lymphohistiocytosis. Pediatr. Blood Cancer.

[13] Griffin G., Shenoi S., Hughes G.C. (2020). Hemophagocytic Lymphohistiocytosis: An Update on Pathogenesis, Diagnosis, and Therapy. Best Pract. Res. Clin. Rheumatol..

[14] Sonoda M., Ishimura M., Eguchi K., Shiraishi A., Kanno S., Kaku N., Inoue H., Motomura Y., Ochiai M., Sakai Y. (2020). Prognostic Factors for Survival of Herpes Simplex Virus-Associated Hemophagocytic Lymphohistiocytosis. Int. J. Hematol..

[15] McKeone D.J., DeMartini T.K.M., Kavanagh R.P., Halstead E.S. (2021). Case Report: Rapid Recognition and Immune Modulation of Secondary HLH Due to Disseminated HSV Infection. Front. Pediatr..

[16] Alidjinou E.K., Dewilde A., Terriou L., Lazrek M., Engelmann I., Hober D. (2015). Persistent Viral DNA Detection in Blood after Primary Herpes Simplex 1 Infection Revealed by Hepatitis with Hemophagocytic Syndrome. J. Clin. Virol..

[17] Cheney-Peters D., Weber D.M. (2019). Herpes Simplex Virus 1 Hepatitis Leading to Liver Failure and Hemophagocytic Lymphohistiocytosis: Case Report and Review of the Literature. Infect. Dis. Clin. Pract..

[18] Cusini A., Günthard H.F., Stussi G., Schwarz U., Fehr T., Grueter E., Meerbach A., Bossart W., Schaer D.J., Rudiger A. (2010). Hemophagocytic Syndrome Caused by Primary Herpes Simplex Virus 1 Infection: Report of a First Case. Infection.

[19] Drori A., Ribak Y., Van Heerden P.V., Meir K., Wolf D., Safadi R. (2015). Hemophagocytic Lymphohistiocytosis Due to Acute Primary Herpes Simplex Virus 1 Infection. J. Clin. Virol..

[20] Freytag M.R., Jørgensen S.E., Thomsen M.M., Al-Mousawi A., Hait A.S., Olagnier D., Bay J.T., Helleberg M., Mogensen T.H. (2022). Postpartum Disseminated Herpes Simplex Virus Type 1 Infection with Hemophagocytic Lymphohistiocytosis and Fulminant Neonatal Herpes Infection. J. Infect. Dis..

[21] Honsig C., Beinhardt S., Tomasits J., Dienes H.P. (2017). Haemophagocytic Lymphohistiocytosis Associated with Fulminant Hepatitis and Multiorgan Failure Following Primary Epstein–Barr Virus and Herpes Simplex Virus Type 1 Infection. BMJ Case Rep..

[22] Halstead E.S., Rajasekaran S., Fitzgerald J.C., Weiss S.L. (2016). Hyperferritinemic Sepsis: An Opportunity for Earlier Diagnosis and Intervention?. Front. Pediatr..

[23] Ikumi K., Ando T., Katano H., Katsuno M., Sakai Y., Yoshida M., Saida T., Kimura H., Sobue G. (2016). HSV-2–Related Hemophagocytic Lymphohistiocytosis in a Fingolimod-Treated Patient with MS. Neurol. Neuroimmunol. Neuroinflamm..

[24] Kojima K., Takahashi N., Yada Y., Koike Y., Matano M., Kono Y., Momoi M.Y. (2012). White-matter Damage in a Neonate with Disseminated Herpes Simplex Virus Infection. Pediatr. Int..

[25] Kurosawa S., Sekiya N., Fukushima K., Ikeuchi K., Fukuda A., Takahashi H., Chen F., Hasegawa H., Katano H., Hishima T. (2019). Unusual Manifestation of Disseminated Herpes Simplex Virus Type 2 Infection Associated with Pharyngotonsilitis, Esophagitis, and Hemophagocytic Lymphohisitocytosis without Genital Involvement. BMC Infect. Dis..

[26] Nagamura N., Ishitobi T. (2017). Hemophagocytic Syndrome Suspected to Be Caused by Herpes Simplex Virus Complicated with Severe Hepatitis during the Immunosuppressive Therapy for Dermatomyositis. Mod. Rheumatol. Case Rep..

[27] Nasser M.F., Sharma S., Albers E., Sharma S., Duggal A. (2018). Pregnancy-Related Hemophagocytic Lymphohistiocytosis Associated with Herpes Simplex Virus-2 Infection: A Diagnostic Dilemma. Cureus.

[28] Noh J.H., Jeong D.Y., Jeon I.S., Kim H.M. (2015). Macrophage Activation Syndrome Triggered by Herpes Viral Infection as the Presenting Manifestation of Juvenile Systemic Lupus Erythematosus. Pediatr. Infect. Vaccine.

[29] Otsubo K., Fukumura A., Hirayama M., Morimoto T., Kato M., Mochizuki H. (2016). Hemophagocytic Lymphohistiocytosis Caused by Systemic Herpes Simplex Virus Type 1 Infection: Successful Treatment with Dexamethasone Palmitate. Pediatr. Int..

[30] Saettini F., Radaelli S., Ocello L., Ferrari G.M., Corti P., Dell’Acqua F., Ippolito D., Foresti S., Gervasini C., Badolato R. (2022). Secondary Hemophagocytic Lymphohystiocytosis in a Rubinstein Taybi Syndrome Patient. Pediatr. Hematol. Oncol..

[31] Schwartz M., O’Brien C., Raya N., Reau N. (2019). Acquired Hemophagocytic Lymphohistiocytosis Associated with Disseminated Herpes Simplex Virus in Immunocompetent Host. ACG Case Rep. J..

[32] Spinner M.A., Ker J.P., Stoudenmire C.J., Fadare O., Mace E.M., Orange J.S., Hsu A.P., Holland S.M. (2016). GATA2 Deficiency Underlying Severe Blastomycosis and Fatal Herpes Simplex Virus–Associated Hemophagocytic Lymphohistiocytosis. J. Allergy Clin. Immunol..

[33] States V.A., Kapp M.E. (2022). Herpes Simplex Virus-1 Triggered Hemophagocytic Lymphohistiocytosis in a Patient with Granulomatosis with Polyangiitis. Autops. Case Rep..

[34] Suzuki N., Morimoto A., Ohga S., Kudo K., Ishida Y., Ishii E. (2009). Characteristics of Hemophagocytic Lymphohistiocytosis in Neonates: A Nationwide Survey in Japan. J. Pediatr..

[35] Takagi Y., Fujita Y., Otaka T., Kano Y., Fukushima K., Sato Y., Yoshihara S. (2023). Postpartum Neonatal Disseminated Herpes Simplex Virus-1 Infection in Which Herpes Simplex Virus-1 Was Detected in Mother’s Breast Milk. Indian J. Pediatr..

[36] Takehara H., Hirohata K., Mutoh H., Irisa C., Kakiuchi S., Nishimura R., Oka A., Takahashi N. (2019). Critically Severe Case of Neonatal Herpes with High Viral Load and Hemophagocytic Syndrome. Tohoku J. Exp. Med..

[37] Vladescu I.A., Browning W.L., Thomsen I.P. (2015). Massive Ferritin Elevation in Neonatal Herpes Simplex Virus Infection: Hemophagocytic Lymphohistiocytosis or Herpes Simplex Virus Alone?. J. Pediatr. Infect. Dis..

[38] Wada Y., Kai M., Tanaka H., Shimizu N., Shimatani M., Oshima T. (2011). Computed Tomography Findings of the Liver in a Neonate with Herpes Simplex Virus-associated Hemophagocytic Lymphohistiocytosis. Pediatr. Int..

[39] Yabushita T., Yoshioka S., Koba Y., Ono Y., Hiramoto N., Tabata S., Itou M., Shimizu N., Tomii K., Ishikawa T. (2017). Successful Treatment of Herpes Simplex Virus (HSV)-1-Associated Hemophagocytic Lymphohistiocytosis (HLH) with Acyclovir: A Case Report and Literature Review. Intern. Med..

[40] Yamada K., Yamamoto Y., Uchiyama A., Ito R., Aoki Y., Uchida Y., Nagasawa H., Kimura H., Ichiyama T., Fukao T. (2008). Successful Treatment of Neonatal Herpes Simplex-Type 1 Infection Complicated by Hemophagocytic Lymphohistiocytosis and Acute Liver Failure. Tohoku J. Exp. Med..

[41] Zhang H., Zhang P., Xiao Z., Gao Y., Han N., He X., Zhang J., Li Y. (2024). Hemophagocytic Lymphohistiocytosis Caused by Herpes Simplex Virus Type 1 in a Young Adult: A Case Report with Literature Review. J. Hematop..

[42] Cole S. (2020). Herpes Simplex Virus. Nurs. Clin. N. Am..

[43] Fatahzadeh M., Schwartz R.A. (2007). Human Herpes Simplex Virus Infections: Epidemiology, Pathogenesis, Symptomatology, Diagnosis, and Management. J. Am. Acad. Dermatol..

[44] Zhu S., Viejo-Borbolla A. (2021). Pathogenesis and Virulence of Herpes Simplex Virus. Virulence.

[45] Nicoll M.P., Hann W., Shivkumar M., Harman L.E.R., Connor V., Coleman H.M., Proença J.T., Efstathiou S. (2016). The HSV-1 Latency-Associated Transcript Functions to Repress Latent Phase Lytic Gene Expression and Suppress Virus Reactivation from Latently Infected Neurons. PLoS Pathog..

[46] Nicoll M.P., Proença J.T., Efstathiou S. (2012). The Molecular Basis of Herpes Simplex Virus Latency. FEMS Microbiol. Rev..

[47] Abdelhay A., Mahmoud A.A., Al Ali O., Hashem A., Orakzai A., Jamshed S. (2023). Epidemiology, Characteristics, and Outcomes of Adult Haemophagocytic Lymphohistiocytosis in the USA, 2006–2019: A National, Retrospective Cohort Study. eClinicalMedicine.

[48] George M.R. (2014). Hemophagocytic Lymphohistiocytosis: Review of Etiologies and Management. J. Blood Med..

[49] Miao Y., Zhang J., Chen Q., Xing L., Qiu T., Zhu H., Wang L., Fan L., Xu W., Li J. (2022). Spectrum and Trigger Identification of Hemophagocytic Lymphohistiocytosis in Adults: A Single-Center Analysis of 555 Cases. Front. Immunol..

[50] Tantawy A.A., Elsherif N.H.K., Elsayed S.M., Ali H.G.A., Makkeyah S.M., Elsantiel H.I.E., De Saint Basile G., Ragab I.A. (2024). Hemophagocytic Lymphohistiocytosis in Egyptian Children: Diagnosis, Treatment Challenges, and Outcome. Expert. Rev. Hematol..

[51] Nienkemper M., Malherbe J., Barrett C. (2020). Haemophagocytic Lymphohistiocytosis: Five Years’ Experience at Tertiary Hospitals in Free State Province, South Africa. S. Afr. J. Crit. Care.

[52] Koumadoraki E., Madouros N., Sharif S., Saleem A., Jarvis S., Khan S. (2022). Hemophagocytic Lymphohistiocytosis and Infection: A Literature Review. Cureus.

[53] Kwak A., Jung N., Shim Y.J., Kim H.S., Lim H.J., Lee J.M., Heo M.H., Do Y.R. (2021). A Retrospective Analysis of Etiology and Outcomes of Hemophagocytic Lymphohistiocytosis in Children and Adults. Yeungnam Univ. J. Med..

[54] Araki S., Shirahata A. (2020). Vitamin K Deficiency Bleeding in Infancy. Nutrients.

[55] Tseng Y.-T., Sheng W.-H., Lin B.-H., Lin C.-W., Wang J.-T., Chen Y.-C., Chang S.-C. (2011). Causes, Clinical Symptoms, and Outcomes of Infectious Diseases Associated with Hemophagocytic Lymphohistiocytosis in Taiwanese Adults. J. Microbiol. Immunol. Infect..

[56] Lim S.H., Park S., Jang J.H., Kim K., Kim H.-J., Kim S.-H., Kang C.-I., Chung D.R., Peck K.R., Lee J. (2016). Clinical Significance of Bone Marrow Hemophagocytosis in Adult Patients with Malignancy and Non-Malignancy-Induced Hemophagocytic Lymphohistiocytosis. Ann. Hematol..

[57] Lin S., Li Y., Long J., Liu Q., Yang F., He Y. (2016). Acute Liver Failure Caused by Hemophagocytic Lymphohistiocytosis in Adults: A Case Report and Review of the Literature. Medicine.

[58] Schram A.M., Comstock P., Campo M., Gorovets D., Mullally A., Bodio K., Arnason J., Berliner N. (2016). Haemophagocytic Lymphohistiocytosis in Adults: A Multicentre Case Series over 7 Years. Br. J. Haematol..

[59] Li J., Wang Q., Zheng W., Ma J., Zhang W., Wang W., Tian X. (2014). Hemophagocytic Lymphohistiocytosis: Clinical Analysis of 103 Adult Patients. Medicine.

[60] Chen T.-Y., Hsu M.-H., Kuo H.-C., Sheen J.-M., Cheng M.-C., Lin Y.-J. (2021). Outcome Analysis of Pediatric Hemophagocytic Lymphohistiocytosis. J. Formos. Med. Assoc..

[61] Nath P., Kabir M.A., Doust S.K., Ray A. (2021). Diagnosis of Herpes Simplex Virus: Laboratory and Point-of-Care Techniques. Infect. Dis. Rep..

[62] Kelesidis T., Humphries R., Terashita D., Eshaghian S., Territo M.C., Said J., Lewinski M., Currier J.S., Pegues D. (2012). Epstein–Barr Virus-associated Hemophagocytic Lymphohistiocytosis in Los Angeles County. J. Med. Virol..

[63] Okazaki K., Imadome K.-I., Nakao H., Miyairi I., Ishiguro A. (2018). Quantitative PCR Assays of Cytomegalovirus and Epstein-Barr Virus in Hemophagocytic Lymphohistiocytosis. Indian J. Pediatr..

[64] Zoref-Lorenz A., Ellis M., Jordan M.B. (2023). Inpatient Recognition and Management of HLH. Hematology.

[65] Jordan M.B., Allen C.E., Greenberg J., Henry M., Hermiston M.L., Kumar A., Hines M., Eckstein O., Ladisch S., Nichols K.E. (2019). Challenges in the Diagnosis of Hemophagocytic Lymphohistiocytosis: Recommendations from the North American Consortium for Histiocytosis (NACHO). Pediatr. Blood Cancer.

[66] Ionescu M.D., Prajescu B., Taras R., Popescu N., Vidlescu R., Smarandoiu M., Rosca L.-E., Enculescu A., Berghea E.C., Toma C.L. (2024). Diagnostic Challenges in Hemophagocytic Lymphohistiocytosis, a Rare, Potentially Fatal Disease: Two Case Studies. J. Clin. Med..

[67] Liew J.W., Jones B.L., Hunter A.J. (2019). Disseminated Herpes Simplex Masquerading as Hemophagocytic Lymphohistiocytosis: A Case Report. Perm. J..

[68] Khandwalla Z., Gupta C., Jhala H. (2021). P0109/#796: Diagnosis of hemophagocytic lymphohistiocytosis in disseminated neonatal herpes simplex virus infection and its influence on outcome. Pediatr. Crit. Care Med..

[69] Averitt G., Al-Rahawan M.M., Levent F. (2019). Neonatal Disseminated Herpes Simplex Virus Infection Triggering Extreme Hyperferritinemia Concerning for Hemophagocytic Lymphohistiocytosis. J. Investig. Med. High Impact Case Rep..

[70] Tabaja H., Kanj A., El Zein S., Comba I.Y., Chehab O., Mahmood M. (2022). A Review of Hemophagocytic Lymphohistiocytosis in Patients with HIV. Open Forum Infect. Dis..

[71] Geng F., Yang M., Zhang X., Zhao H., Zhou D., Hu J. (2023). Typical Hemophagocytic Syndrome Associated with Cytomegalovirus Infection in an Immunocompetent Patient: A Case Report and Literature Review. J. Zhejiang Univ. Sci. B.

[72] Rolsdorph L.Å., Mosevoll K.A., Helgeland L., Reikvam H. (2022). Concomitant Hemophagocytic Lymphohistiocytosis and Cytomegalovirus Disease: A Case Based Systemic Review. Front. Med..

[73] Van Der Ven A.J.A.M., Netea M.G., Van Der Meer J.W.M., De Mast Q. (2015). Ebola Virus Disease Has Features of Hemophagocytic Lymphohistiocytosis Syndrome. Front. Med..

[74] Marsh R.A. (2018). Epstein–Barr Virus and Hemophagocytic Lymphohistiocytosis. Front. Immunol..

[75] Oh E.-J., Yoon J.-H., Park K.H., Bae H.J., Yun S.J., Min G.J., Park S.-S., Park S., Lee S.-E., Cho B.-S. (2021). Natural-Killer Cell Cytotoxicity as a Diagnostic and Prognostic Marker for Adult Patients with Secondary Hemophagocytic Lymphohistiocytosis: A Prospective Phase II Observational Study. Ther. Adv. Hematol..

[76] Ramos-Casals M., Brito-Zerón P., López-Guillermo A., Khamashta M.A., Bosch X. (2014). Adult Haemophagocytic Syndrome. Lancet.

[77] Gupta A., Weitzman S., Abdelhaleem M. (2008). The Role of Hemophagocytosis in Bone Marrow Aspirates in the Diagnosis of Hemophagocytic Lymphohistiocytosis. Pediatr. Blood Cancer.

[78] See K.C. (2024). Dengue-Associated Hemophagocytic Lymphohistiocytosis: A Narrative Review of Its Identification and Treatment. Pathogens.

[79] Lee H.-Y., Huang Y.-C., Lin T.-Y., Huang J.-L., Yang C.-P., Hsueh T., Wu C.-T., Hsia S.-H. (2010). Primary Epstein-Barr Virus Infection Associated with Kikuchi’s Disease and Hemophagocytic Lymphohistiocytosis: A Case Report and Review of the Literature. J. Microbiol. Immunol. Infect..

[80] Gholam C., Grigoriadou S., Gilmour K.C., Gaspar H.B. (2011). Familial Haemophagocytic Lymphohistiocytosis: Advances in the Genetic Basis, Diagnosis and Management. Clin. Exp. Immunol..

[81] Allen C.E., McClain K.L. (2015). Pathophysiology and Epidemiology of Hemophagocytic Lymphohistiocytosis. Hematology.

[82] Canna S.W., Marsh R.A. (2020). Pediatric Hemophagocytic Lymphohistiocytosis. Blood.

[83] Carvelli J., Piperoglou C., Farnarier C., Vely F., Mazodier K., Audonnet S., Nitschke P., Bole-Feysot C., Boucekine M., Cambon A. (2020). Functional and Genetic Testing in Adults with HLH Reveals an Inflammatory Profile Rather than a Cytotoxicity Defect. Blood.

[84] Chinnici A., Beneforti L., Pegoraro F., Trambusti I., Tondo A., Favre C., Coniglio M.L., Sieni E. (2023). Approaching Hemophagocytic Lymphohistiocytosis. Front. Immunol..

[85] Lehmberg K., Sprekels B., Nichols K.E., Woessmann W., Müller I., Suttorp M., Bernig T., Beutel K., Bode S.F.N., Kentouche K. (2015). Malignancy-associated Haemophagocytic Lymphohistiocytosis in Children and Adolescents. Br. J. Haematol..

[86] Sieni E., Cetica V., Hackmann Y., Coniglio M.L., Da Ros M., Ciambotti B., Pende D., Griffiths G., Aricã2 M. (2014). Familial Hemophagocytic Lymphohistiocytosis: When Rare Diseases Shed Light on Immune System Functioning. Front. Immunol..

[87] Bracaglia C., Prencipe G., De Benedetti F. (2017). Macrophage Activation Syndrome: Different Mechanisms Leading to a One Clinical Syndrome. Pediatr. Rheumatol..

[88] Brisse E., Wouters C.H., Andrei G., Matthys P. (2017). How Viruses Contribute to the Pathogenesis of Hemophagocytic Lymphohistiocytosis. Front. Immunol..

[89] Jordan M.B., Allen C.E., Weitzman S., Filipovich A.H., McClain K.L. (2011). How I Treat Hemophagocytic Lymphohistiocytosis. Blood.

[90] Imashuku S., Kuriyama K., Teramura T., Ishii E., Kinugawa N., Kato M., Sako M., Hibi S. (2001). Requirement for Etoposide in the Treatment of Epstein-Barr Virus–Associated Hemophagocytic Lymphohistiocytosis. J. Clin. Oncol..

[91] Chellapandian D., Das R., Zelley K., Wiener S.J., Zhao H., Teachey D.T., Nichols K.E., EBV-HLH Rituximab Study Group (2013). Treatment of E Pstein B Arr Virus-induced Haemophagocytic Lymphohistiocytosis with Rituximab-containing Chemo-immunotherapeutic Regimens. Br. J. Haematol..

[92] Kumar B., Aleem S., Saleh H., Petts J., Ballas Z.K. (2017). A Personalized Diagnostic and Treatment Approach for Macrophage Activation Syndrome and Secondary Hemophagocytic Lymphohistiocytosis in Adults. J. Clin. Immunol..

[93] Bichon A., Bourenne J., Allardet-Servent J., Papazian L., Hraiech S., Guervilly C., Pauly V., Kaplanski G., Mokart D., Gainnier M. (2021). High Mortality of HLH in ICU Regardless Etiology or Treatment. Front. Med..

[94] Jongdee P., Julamanee J., Rattarittamrong E., Mukura S., Wanitpongpun C., Deoisares R., Surawong A., Chajuwan T., Chanswangphuwana C. (2024). Prognostic Factors of Adult Hemophagocytic Lymphohistiocytosis and Clinical Utility of HLH-2004 Diagnostic Criteria and HScore: A Real-World Multicenter Study from Thailand. Acta Haematol..

[95] Parikh S.A., Kapoor P., Letendre L., Kumar S., Wolanskyj A.P. (2014). Prognostic Factors and Outcomes of Adults with Hemophagocytic Lymphohistiocytosis. Mayo Clin. Proc..

[96] Oto M., Yoshitsugu K., Uneda S., Nagamine M., Yoshida M. (2015). Prognostic Factors and Outcomes of Adult-Onset Hemophagocytic Lymphohistiocytosis: A Retrospective Analysis of 34 Cases. Hematol. Rep..

